# Pyrolysis of Polystyrene Waste: A Review

**DOI:** 10.3390/polym13020225

**Published:** 2021-01-11

**Authors:** Ibrahim M. Maafa

**Affiliations:** Chemical Engineering Department, College of Engineering, Jazan University, Jazan 45142, Saudi Arabia; imoaafa@jazanu.edu.sa

**Keywords:** polystyrene, pyrolysis, fuel oils

## Abstract

The manufacturing of polystyrene around the globe has escalated in the past years due to its huge applications in various areas. The perpetual market needs of polystyrene led the polystyrene wastes accretion in the landfill causing environmental deterioration. The soaring need for polystyrene also led to the exhaustion of petroleum, a non-renewable energy source, as polystyrene is a petroleum-derived product. Researchers from around the world have discovered a few techniques to take care of the polystyrene scraps, namely recycling and energy recovery techniques. Nevertheless, there are demerits involved with recycling techniques, such as they call for huge labor expenses in the separation process and cause water pollution, thereby decreasing the process sustainability. Owing to these demerits, the researchers have focused their attention on the energy recovery technique. Since petroleum is the main ingredient of polystyrene synthesis, the restoration of liquid oil from polystyrene via the pyrolysis method is a promising technique as the recovered oil has greater calorific value as compared to commercially available fuel. The present paper surveys the pyrolysis technique for polystyrene and the important process parameters that control the end product, like oil, gas, and char. The chief process parameters that are discussed in this review paper include the type of reactors, temperature, residence time, pressure, catalyst types, type of fluidizing gases, and their flow rate. A more recent technique of utilizing a solvent to perform pyrolysis and the effect of various process conditions on the product yield have been discussed. Apart from this, various outlooks to optimize the liquid oil recovery from polystyrene are also reviewed.

## 1. Introduction

For the past fifty years, plastic has played a crucial role in upgrading the human society [1]. It has imparted impulse in the development of numerous sectors like packaging, electronics, automobiles, medical, construction, etc. [1]. Due to the swift surge in the global population, the market need for plastics has risen. Owing to this, the total manufacturing of plastic throughout the world attained approximately 359 million tonnes in 2018 [2]. The regular increase in the requirement of plastic led to plastic waste accretion every year. Based on 2013 data, plastic scrap amounting to approximately 33 million tonnes was produced in the US [3]. As far as Europe is concerned, around 25 million tonnes of plastic finally turned out as scrap in the year 2012; out of this waste plastic approximately 38% accreted in the landfill, 26% was recycled whilst 36% was recovered through energy recovery technique [4]. These data signify that the quantity of plastic scrap that has accumulated in the landfill is too large. As a fact, natural degradation of plastics may take billions of years since its molecules constituting carbon, hydrogen, nitrogen, and chlorine are attached through very strong bonds [5]. Consequently, the regular accretion of plastic in the landfill poses a severe environmental hazard. To curtail the accumulation of plastic in the landfill, researchers developed techniques to recycle it [6]. However, recycling the plastic turns out to be a cumbersome and costly process since it requires excessive labor to sort out plastics and also results in water contamination [7]. Sorting of plastics is required before recycling as different plastic polymers have their characteristics such as resin compound they are constituted of, color, and transparency. The energy recovery technique is another method that transforms plastics directly into useful forms of energy and chemicals for industries since plastics are mainly derived from petroleum.

One of the most important and widely used plastic is polystyrene (PS) which is cheap and hard. It is transparent but can be colored by introducing colorants. It is heat resistant, lighter in weight and, exhibits good strength and durability that make this polymer fit for a variety of applications. The range of applications includes packaging, toys, and household items like computer housing and kitchen appliances, etc. PS are available in expanded and solid forms, and both of these forms are recyclable. However, expanded polystyrene foam waste loses its foam characteristics once recycled. It is possible to re-gas the recovered polystyrene, but it makes the product costlier than the virgin material. Thus, it is utilized in solid state in molding processes.

Expanded and solid PS scraps have been easily recycled to extruded plastic lumber. This extruded polystyrene (XPS) has been widely employed to construct windows systems, roof trusses and flooring of buildings and houses. Recycled PS is employed to manufacture plant pots, pens, and pencils, etc., whereas PS foam is used extensively as an insulator. Expanded PS can also be utilized as substrates to produce poly-electrolytes which display better flocculation characteristics compared to that of commercial Praestol 2515 [8].

Recycled and virgin PS are being employed to produce a material with practically the same properties as that of wood. This novel material exhibits striking resemblance to wood in respect of physical appearance, density, and structural properties, making it a suitable candidate that can replace wood in furniture and construction industry. Non-foam polystyrene materials also known as high impact polystyrene (HIPS), oriented polystyrene (OPS), Styrofoam, post-industrial products and post-consumer products are accepted as recyclable materials [9].

A large portion of the PS waste is disposed as solid pollutant in the developing countries. Moreover, the increased use of PS in electronic gadgets has rapidly enhanced the waste from electrical and electronic equipment (WEEE) which makes the situation more problematic. In addition, a large amount of expanded PS is routinely disposed at wholesale markets, supermarkets, departmental stores, restaurants, and shops, as well as machine manufacturing factories. It is later gathered by the recycling agents as a recycling resource. However, a number of issues have to be taken into consideration by the industries before recycling PS, such as, eco-friendliness, corporate social responsibility, hygienic prospective, and traceability. Prior to recycling, the waste materials must be washed properly to remove any stuck food or dust particles, the metallic caps and glass jars must be separated from the raw materials, and oversized commodities must be crushed so as to fit them into the bin and transporting truck easily. The volume of the PS waste can then be reduced by various techniques like dissolving into solvent, heating, and pulverizing.

The conventional method for the treatment of polystyrene is pyrolysis, which involves thermal decomposition without air to produce pyrolysis oils or gases and monomer or other valuable chemicals [10]. The pyrolysis process requires proper reactor and catalyst selection that control the pyrolysis temperature and end-products. A lot of researchers have devoted their study on the investigation of influence of reactor design (such as, batch-type, fixed-bed, continuous flow and pressure reactors) and material [11,12,13,14,15]. The utilization of solvents, various plastic blends and additives for carrying out pyrolysis is also found to be a promising method to optimize the yield of liquid products [16,17]. A lot of work has been focused on the pyrolysis of PS employing acidic and basic catalysts to enhance the yield and selectivity of products, and achieve a cheap pyrolysis method [18,19,20,21,22]. This review article deals with all the above issues, and presents an exhaustive up-to-date survey of the various methods employed so far to carry out pyrolysis of PS waste. Thus, it will be extremely useful for the scientists who are researching in this broad area to identify the fundamental issues and the major contributors. The prime and fundamental purpose of presenting this review article is to provide researchers and readers with a legible and unambiguous synthesis of the best resources available in the literature pertinent to the pyrolysis of PS waste.

## 2. Pyrolysis of Polystyrene

Polystyrene (PS) is made up of styrene monomers obtained from the liquid petrochemical. It comprises of a long hydrocarbon chain with phenyl groups linked to alternating carbon atoms. Presently, most of the polystyrene waste is disposed to the landfills and not recycled because of the problems encountered while separating and cleaning polystyrene. This creates a threat to the environment and causes damage to human beings, wild and aquatic life. Thus, it becomes very important to convert polystyrene into valuable products through pyrolysis. The pyrolysis process involves heating plastic at a high temperature of 300–900 °C in the absence of oxygen, wherein the long polymeric chains get decomposed into useful low molecular weight liquid and gaseous products. Oil, gas, and char are the three basic byproducts that are finally produced during pyrolysis. These byproducts are very useful in the production and refining industries. The produced liquid oil offers wide applications as fuel in furnaces, turbines, boilers, and diesel engines without any further treatment.

Pyrolysis is considered to be very flexible since involved parameters can be altered easily to obtain an optimum yield of the product. In contrast to the recycling process, pyrolysis does not cause water pollution and hence is considered eco-friendly technology [23]. Pyrolysis process had received a lot of attention from the scientific community since this process can finally produce liquid oil up to 80 wt.% at an easily achievable temperature of about 500 °C [24]. The pyrolysis of PS in a batch pressurized autoclave reactor had been reported by Onwudili et al. [25]. They carried out pyrolysis for one hour at a temperature range of 300–500 °C with a rate of heating at 10 °C/min and variation of ambient pressure from 0.31 MPa to 1.6 MPa. They noted that the pyrolysis of PS resulted in the production of approximately 97.0 wt.% liquid oil and maximum gas production of 2.5 wt.% at an optimum temperature of 425 °C. Such a high production of liquid oil was also reported by Liu et al. [12] during the pyrolysis of PS employing a fluidized bed reactor at a temperature range of 450–700 °C. The observed record production of liquid oil was 98.7 wt.% at 600 °C. However, Demirbas at al. [26] reported that liquid oil production decreased to 89.5 wt.% when the pyrolysis was carried out in a batch reactor at 581 °C. Thus, it is not recommended to carry out pyrolysis of PS at a temperature exceeding 500 °C to achieve maximum liquid oil production.

To sum up, the production of liquid and gaseous by-products from the pyrolysis of polystyrene relies mainly on the reaction conditions. Catalytic pyrolysis using suitable catalysts can sway the yielded products and their distribution, in addition to the reduced reaction temperature. This advantage leads to the high contents of products having higher commercial value [27,28]. Polystyrene could be converted into styrene over catalysts by simple thermal cracking at relatively low temperatures. The pyrolysis of polystyrene mainly depends on the reaction conditions such as temperature, reaction time, reactor type, the presence of catalysts, etc. At low temperatures, products mainly consist of liquid compounds (mono aromatic). At higher temperatures, gas and coke yields are higher and the liquid fraction has significant aromatics (dimer, trimer) [27,28,29,30]. The pyrolysis of polystyrene had been previously investigated at a comparatively lower temperature ranging from 370 °C to 400 °C employing a batch-type stirred reactor. The main products were single aromatic species (styrene ca. 70 wt.%, α-methyl styrene, toluene) and double aromatic species (1,3-diphenylpropane and 1,3-diphenylbutene) [29]. The pyrolysis of polystyrene in a fluidized-bed reactor at 550 °C generated the residue that contains 19–20% for oil yields and 10–11% for styrene monomers [31,32]. Thermal decomposition of expanded polystyrene in a pebble bed reactor at 500 °C produced 91.7% of liquid products while yielding 85.5% of styrene using ceramic pebbles [11]. Several studies have been conducted on catalytic pyrolysis of polystyrene over different solids like metallic oxides (silica-alumina, $K_{2}O,\;\mathrm{CaO},\;\mathrm{BaO},{\;\mathrm{SiO}}_{2},{\;\mathrm{Al}}_{2}O_{3},\mathrm{CuO}/{\mathrm{Al}}_{2}O_{3}$, alumina or silica supported transition metals) [19,30,33,34], zeolites (HNZ, ZSM−5, FCC, clinoptilolite, Al−MCM−41, dealuminated HY) [32,33,34,35], mesoporous materials ($K_{2}O/\mathrm{Si}-\mathrm{MCM}-41,{\;K}_{2}O-\mathrm{BaO}/\mathrm{MCM}-4,\;\mathrm{MCM}-41$ from natural sepiolite) [36,37], and clays (halloysite, albite, montmorillonite, and pyrophyllite) [38,39]. The use of catalysts in the pyrolysis of polystyrene waste determines the good selectivity for the formation of costly hydrocarbons and the use of lower temperatures. Thus, the search for “cheap catalysts” for pyrolysis is of particular interest from an industrial and economical point of view [40]. The catalyst cost, type, and its amount represent key factors in determining the economy of catalytic processes because, in a continuously operating plant, it is necessary to have a large amount of catalyst. Mostly, natural catalysts are employed since their textural properties, such as surface area, particle size, and pore size distribution play an important role in end-product distribution. In this review, we will focus on the influence of temperature and type of catalysts on the yields and distribution of end products in thermal and catalytic pyrolysis. Furthermore, we have also surveyed a recent method to conduct the pyrolysis process in the presence of a solvent in pursuit to optimize the quality and quantity of oil produced [41]. Table 1 summarizes the main advantages of the various methods employed for the pyrolysis of PS.

## 3. Process Parameters

There are certain crucial process parameters of pyrolysis that govern the production of end products. These parameters include temperature, pressure, residence time, catalysts, type of reactors, and type of fluidizing gas and its flow rate. We will review the effect of these operating parameters and their control in the following subsections thoroughly.

### 3.1. Temperature

The most important process parameter in pyrolysis is temperature since it governs the cracking reaction of the polymeric chain of polystyrene. PS degrades at the lowest temperature in the pyrolysis process from among the variety of plastics. The pyrolysis of PS in a batch reactor was reported by Onwudili et al. [25], who observed that till 300 °C no reaction took place. Nevertheless, increasing the temperature to 350 °C degraded the entire PS into dark-colored liquid oil of very high viscosity. The maximum quantity of liquid oil was produced at 425 °C. Increasing the temperature to 581 °C decreased the quantity of liquid oil produced while increasing the production of gaseous products [26]. Thus, it is noteworthy that the temperature range for the degradation of PS during pyrolysis is around 350–500 °C. From the above discussion, it can be well established that the nature of the byproducts formed during pyrolysis strongly depends on temperature. If the desired product is gaseous or char then a higher temperature above 500 °C is recommended, while if liquid oil is the preferred case then a relatively lower temperature range of 300–500 °C is recommended.

### 3.2. Reactors

The type of reactor decides the efficiency of the blending of polystyrene with the catalysts, residence time, heat transfer rate, and efficiency of reaction to obtain desired byproducts. Mostly, the pyrolysis of plastics is carried out in the lab in batch, semi-batch, and continuous flow reactors. The following subsections address their relative merits and demerits.

#### 3.2.1. Batch Reactor

A batch reactor acts as a closed system wherein there is no inflow or outflow of reactants and byproducts during the reaction. Its main advantage is that it can yield high conversion efficiency by allowing the reactants to stay in the reactor for a longer period. However, there are certain downsides of this reactor such as the production of different byproducts in every batch, high labor expenses per batch, and unsuitability for mass production [42]. On the contrary, in a semi-batch reactor, the supply of reactant and byproduct extraction can be done simultaneously.

Thus, regarding the reaction selectivity, the semi-batch reactor offers an advantage of flexibility in reactant addition over time. However, the semi-batch reactor has a similar drawback of high labor cost as in the case of batch reactor making it fitter for production at a small scale. Instead of the above-discussed downsides associated with the batch or semi-batch reactors, researchers prefer them to carry out laboratory-scale experiments because of the simplicity in their design and easy control of the operating parameters [51,52,53,54,55,56,57,58,59,60].

Both the thermal and catalytic pyrolysis is carried out in a batch or semi-batch reactor usually within a temperature range of 300–800 °C. Catalysts are mixed with the polystyrene during reaction inside the batch reactor to increase the production of hydrocarbon and useful products. However, there is a disadvantage associated with the catalyst addition in a batch reactor which is the high propensity for the formation and accumulation of coke on the catalyst surface diminishing the catalyst efficiency with the progress of time and high residual leftover in the process. Also, it is a major challenge to separate the residues from the catalyst at the end of the reaction. To sum up, batch or semi-batch reactors are not fit for catalytic pyrolysis and large-scale production since feedstock recharging in these reactors requires high operating cost and hence, they are preferred only for conducting experiments [61].

**Figure 1:**
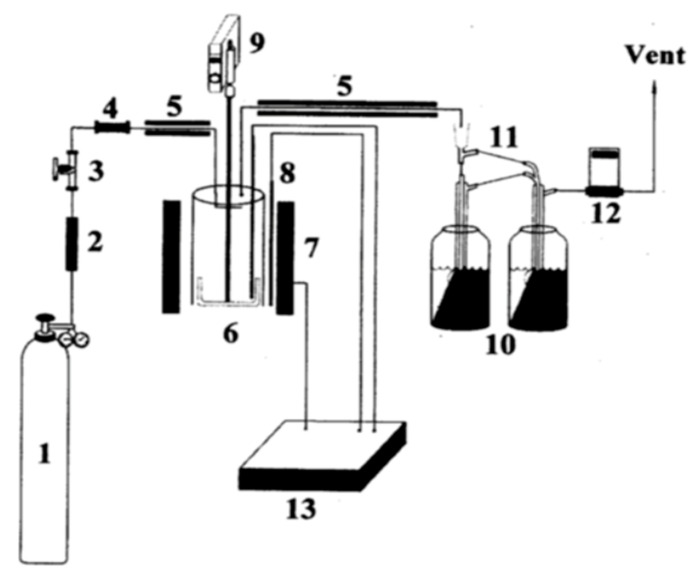
Schematic diagram of the batch-type stirred reactor employed for the pyrolysis of polystyrene. The various components of this reactor are as follows: (**1**) N2 bomb; (**2**) Silica gel trap; (**3**) Control valve; (**4**) Check valve; (**5**) Heating band; (**6**) Batch reactor; (**7**) Electric furnace; (**8**) Chromel-Alumel thermocouple; (**9**) Agitator; (**10**) Liquid N2 trap; (**11**) Condenser; (**12**) Gas flow monitor; (**13**) Temperature controller. Reproduced with permission from [29].

Kim et al. [29] carried out isothermal pyrolysis of PS in a relatively low-temperature range of 370–400 °C in a batch-type stirred reactor, as shown in Figure 1. The reactor was fabricated from stainless steel and had 124 mm internal diameter, 134 mm external diameter, and 205 mm height. To minimize the temperature profile in the reactor, a mechanical stirrer was installed in it. For maintaining an isothermal and homogeneous condition, the stirrer was rotated at a speed of 80 rpm. To condense the formed gases, two Pyrex glass condensers were attached to the reactor. The temperature of the reactor was monitored with the aid of a proportional–integral–derivative (PID) temperature controller accoutered with a programmable device.

**Figure 2:**
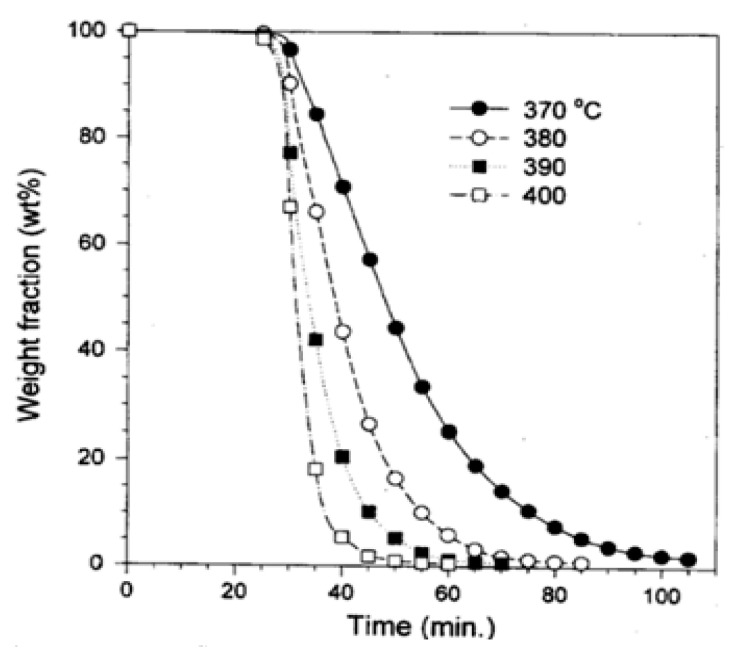
The weight fraction of yield at various temperatures [29].

**Figure 3:**
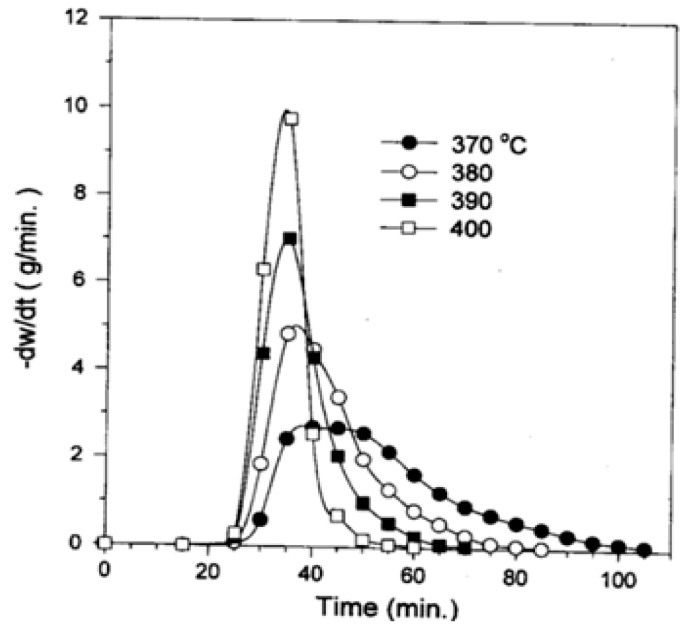
Variation of pyrolysis rate with increasing time [29].

Figure 2 displays the experimental results from the pyrolysis of PS beyond 25 min. heating at various temperatures. The yield of pyrolysis increased with the rise in the reactor temperature. The variation of the rate of pyrolysis with time is displayed in Figure 3. The peak pyrolysis rate was observed approximately at 40 min of heating for the experiment conducted at 370 °C, while the peak rate was attained after 35 min of heating at 380, 390, and 400° C.

Table 2 summarizes the yields of liquid oil and gaseous products from the pyrolysis of PS at various temperatures. As the temperature increased, the gaseous products yield increases.

Table 3 summarizes the temperature required to optimize the yield of liquid oil from the pyrolysis of PS carried out in batch reactors working under different configurations and conditions. Other controlling parameters comprised of the pressure, heating rate and the residence time of the charge inside the reactor. All the experiments were carried out employing nitrogen gas as the fluidizing medium. It should be noted that catalysts have also been utilized in some of the experiments. These catalytic effects will be discussed in a more detailed way in later sections.

#### 3.2.2. Fixed and Fluidized Bed Reactor

**Figure 4:**
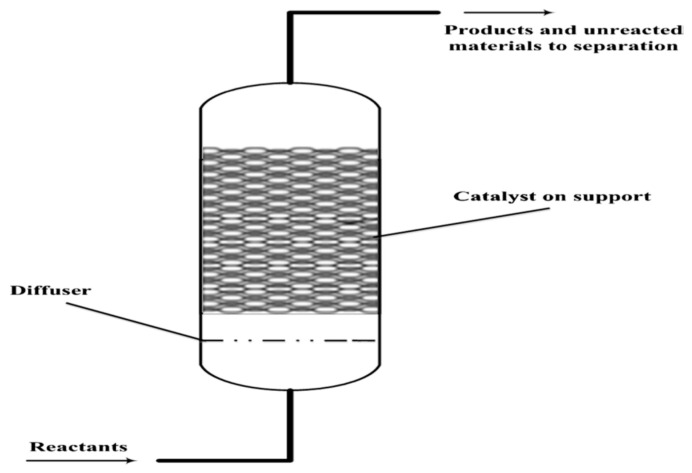
Diagram of the fixed-bed reactor [68].

In the fixed bed reactor, the catalyst is normally provided in the form of pallets and packed as a static bed as displayed in Figure 4. It is simple in design, but it has a constraint that irregular shape and size of polystyrene scrap cannot be fed into the reactor. Various researchers had employed a fixed bed reactor for the pyrolysis of polystyrene [43,63,64,65,66,67]. These reactors are mostly employed as secondary reactors wherein the byproduct from the primary pyrolysis normally in the form of liquid and gas is usually fed into the fixed bed reactor [42]. However, these two-step pyrolysis processes are not economical and the final products produced are quite similar to those produced in a single-step process.

**Figure 5:**
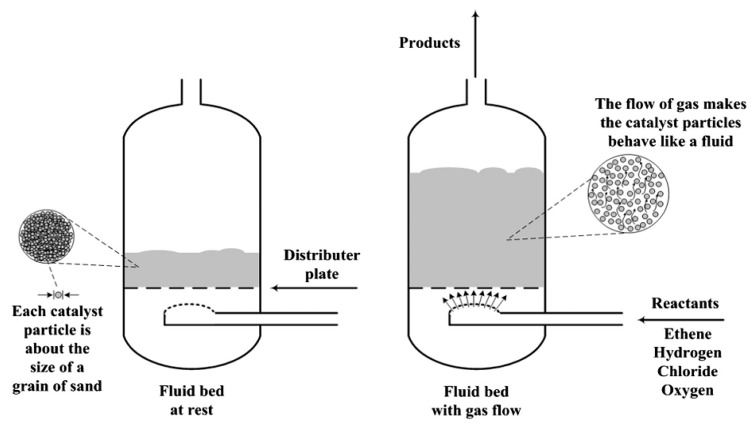
Diagram of fluidized bed reactor [42].

To overcome the drawbacks related to the fixed bed reactors, researchers developed a fluidized bed reactor. In a fluidized bed reactor, the catalyst is uniformly distributed on a distributor plate and the fluidizing gas is blown through it, thus carrying the particles in a fluidized state, as depicted in Figure 5. In this way, the catalytic efficiency increases since the catalyst blend well with the charge fluid providing a large surface area to carry out the reaction [69]. This also enhances the heat transfer rate during the pyrolysis. A fluidized bed reactor is also better than a batch reactor since feedstock charging in it is not needed frequently and the process does not need to restart repeatedly. In conclusion, considering the traditional design scale, a fluidized bed reactor is regarded as the best reactor that can be employed in the pilot plant because of its low operating cost and large-scale operation [12].

**Figure 6:**
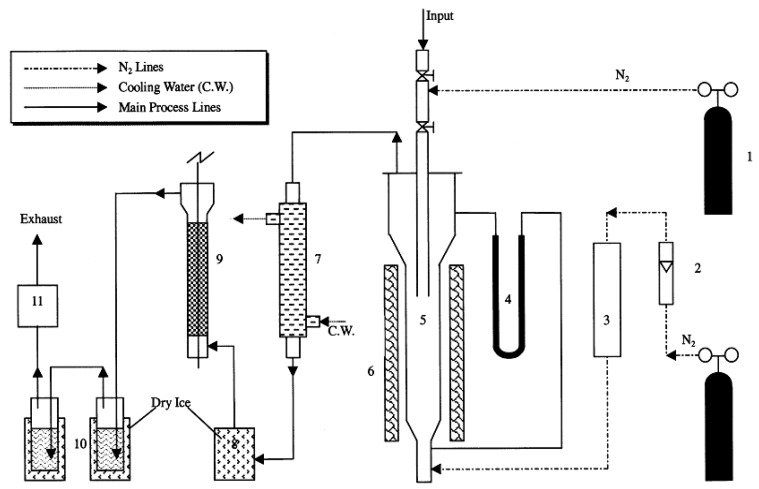
Schematic diagram of the fluidized-bed reactor apparatus: (**1**) Nitrogen bottle; (**2**) Rotameter; (**3**) Gas preheater; (**4**) U-shaped pressure gauge; (**5**) Fluidized-bed reactor; (**6**) Clamshell furnace; (**7**) Primary condenser; (**8**) Dry ice condenser; (**9**) Electrostatic demister; (**10**) Bubble adsorption unit; (**11**) Gas sample bag [12].

Figure 6 displays the schematic diagram of the fluidized bed reactor setup, as reported by Liu et al. [12]. The reactor was designed to operate at a temperature range of 400–800 °C, with the fluidization zone of 150–200 mm height and 60 mm diameter. To make sure that the fluidization of the system happens uniformly, the angles of cone-shaped components were selected meticulously considering the diffusibility of the fluidizing gas. The reactor was loaded with 20/40 mesh quartz sand which acts as the fluidization and heat supplying medium. Nitrogen was employed as the fluidizing gas and an external electric clamshell furnace having a rating of 2.5 kW was employed to heat the sand particles in the reactor. Before the experiment, the reactor was made oxygen-free by flowing nitrogen into it.

The feedstock comprised of disposed PS dishware and packaging materials which were crushed, ground and sieved to around 3 × 4 mm particle size, and then fed into the reactor manually through the feed tube. The batch weight of PS feed was 10 g each time. The PS particles were properly blended with the hot sand in the reactor, and further heated to the already set temperature and pyrolyzed. The gaseous products were rapidly separated from the solid phase and allowed to exit from the reactor through the top outlet.

**Figure 7:**
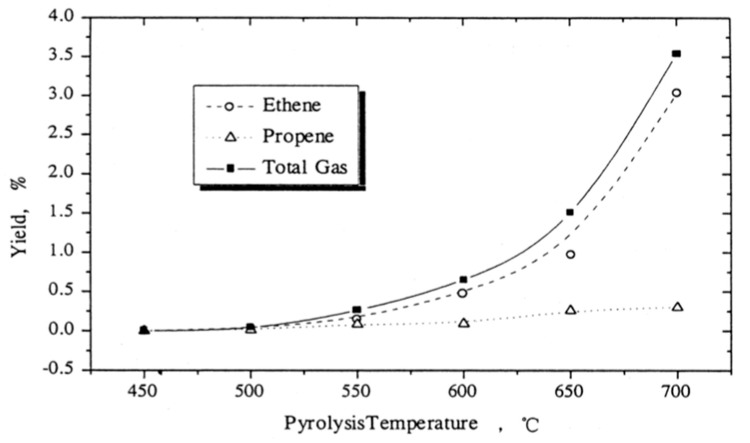
Variation of cracking gas yield with pyrolysis temperature [12].

Liu et al. [12] reported that the major gas components produced after the pyrolysis of PS were ethylene and propylene. Figure 7 displays the variation of gaseous products yield with the reactor temperature. It was observed that gaseous product yield increased with increasing temperature, especially in the case of ethylene.

**Figure 8:**
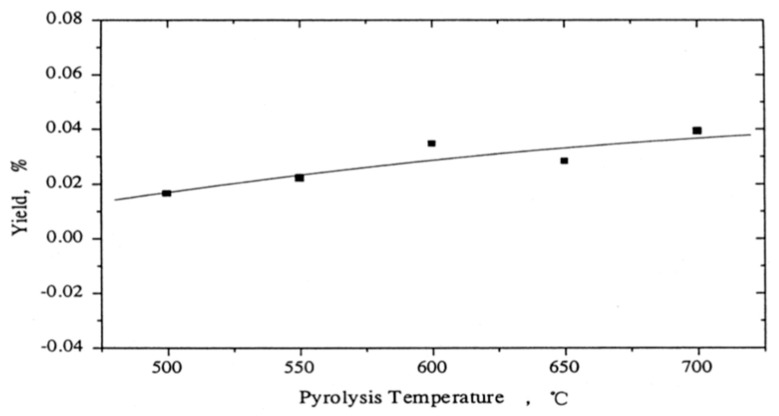
Variation of coke yield with pyrolysis temperature [12].

The coke produced was determined from the coke deposited on the surface of quartz sand, and calculated by the difference in its weight before and after the experiment. Figure 8 shows the variation of coke yield with pyrolysis temperature, wherein it can be observed that coke formation increased with increasing temperature.

**Figure 9:**
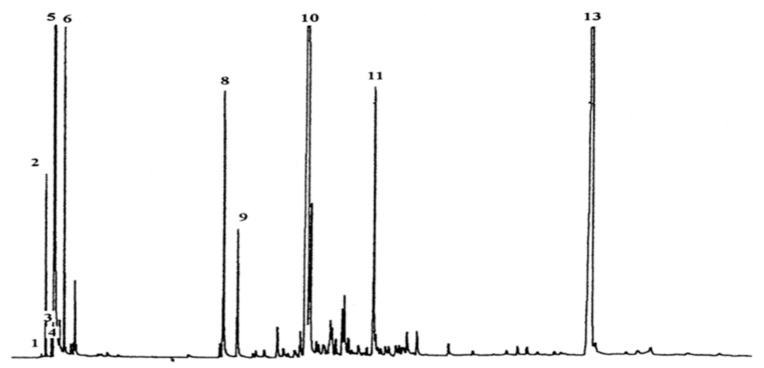
Chromatogram of liquid yield from PS pyrolysis [12].

The main components of liquid yield from PS pyrolysis as obtained from chromatography (see Figure 9) were styrene monomer, dimer, and trimer. Some monoaromatics were also found like toluene, benzene, ethylbenzene, and α-Methylstyrene as summarized in Table 4.

For simplicity, all the liquid products were categorized into three groups based on their boiling point:

- Low boiling fraction (BP less than 200 °C): G1 (styrene and monoaromatics)
- Medium boiling fraction (BP greater than 200 °C and less than or equal to 350 °C): G2(dimer and others)
- High boiling fraction (BP greater than 350 °C): G3 (trimer and others)

**Figure 10:**
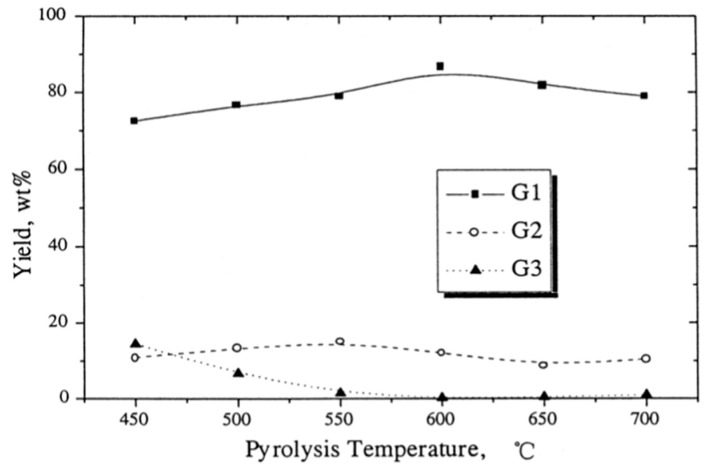
The variation of liquid yield percentage with pyrolysis temperature [12].

From Figure 10, we notice that within the temperature range of 450–700 °C, the G3 fraction yield decline sharply from 14.7 wt.% to 0.11 wt.%with the increase in temperature. However, the G1 fraction yield increased to a maximum value of 86.6 wt.% at 600 °C and further beyond 600 °C decreased due to condensation. The G2 fraction yield showed small variations of about 10 wt.% during the whole temperature range.

#### 3.2.3. Conical Spouted Bed Reactor (CSBR)

Conical spouted bed reactor (CSBR) allows better blending of charge and treat large particles with density disparity [42]. Many researchers successfully employed CSBR for the catalytic cracking of plastics [44,48,70,71,72,73]. Olazar et al. [71] declared that CSBR has lower debilitation and bed segregation in comparison to a bubbling fluidized bed reactor. Also, it provides a higher heat transfer rate and can handle sticky solids efficiently that was difficult to handle in a fluidized bed reactor. The spouted bed design is befitting to carry out low-temperature pyrolysis to produce wax. It has been observed that the production of waxes reduced with increasing temperature since at higher temperatures more waxes can be degraded into a liquid or gaseous product. Various technical issues have been encountered while operating this reactor like a problem in catalyst feeding, catalyst entrainment, and product accumulation at the end of pyrolysis [42]. Moreover, the complicated design of this reactor demands many pumps to be installed in the reactor raising the operating cost exorbitantly high.

**Figure 11:**
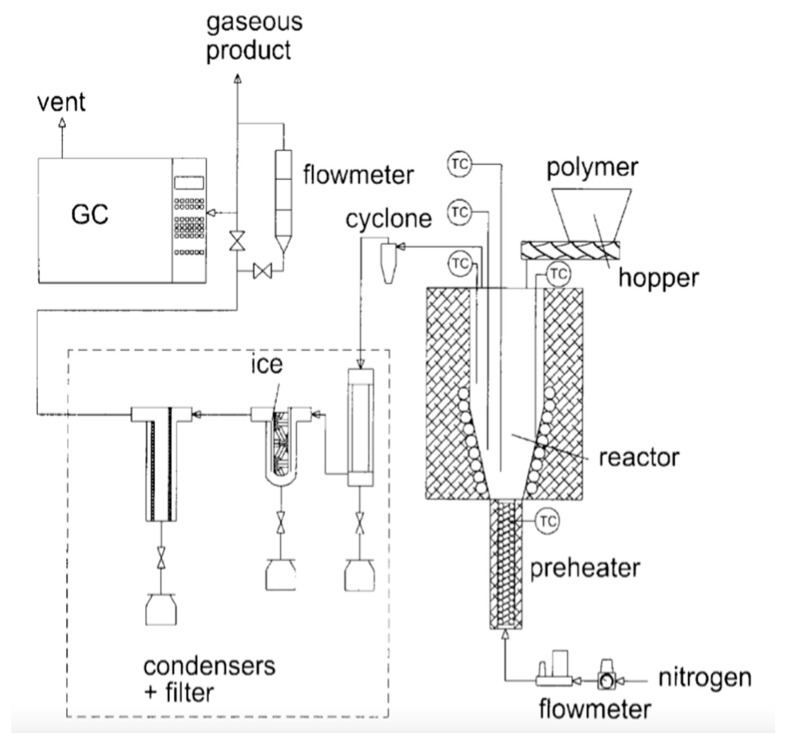
Schematic diagram of conical spouted bed reactor (CSBR). Reproduce with permission from Aguado et al. [73].

Aguado et al. [73] carried out the pyrolysis of polystyrene in a CSBR in a temperature range of 450–550 °C. Figure 10 displays the schematic of the laboratory setup of CSBR. The PS is fed through the inlet tube placed over the annular region of the bed. The temperature was monitored via three thermocouples (TCs) positioned at various radial spots of the reactor and can move freely vertically allowing temperature recording at any position of the reactor. The feedstock was heated to reaction temperature via two electrical resistances enveloped by ceramic material, as shown in Figure 11. One resistance was employed in the tube to heat the inert gas before the introduction in the reactor and the second one was employed in the conical region of the reactor. These two regions were thermally insulated and the temperature at these two locations was measured by two thermocouples.

The flow of pyrolysis by-products was carried by the inert gas to the condensing system comprising of a cooling element, salt-water ice trap, and a 25 µm sintered steel filter. The gaseous products from the product stream were analysed by gas chromatography (Hewlett-Packard 6890) employing a BPX5 capillary column of 50 m. Within the operating conditions, all the PS pyrolysis by-products were found to be present in the gaseous stream. The product yield is summarized in Table 5.

**Figure 12:**
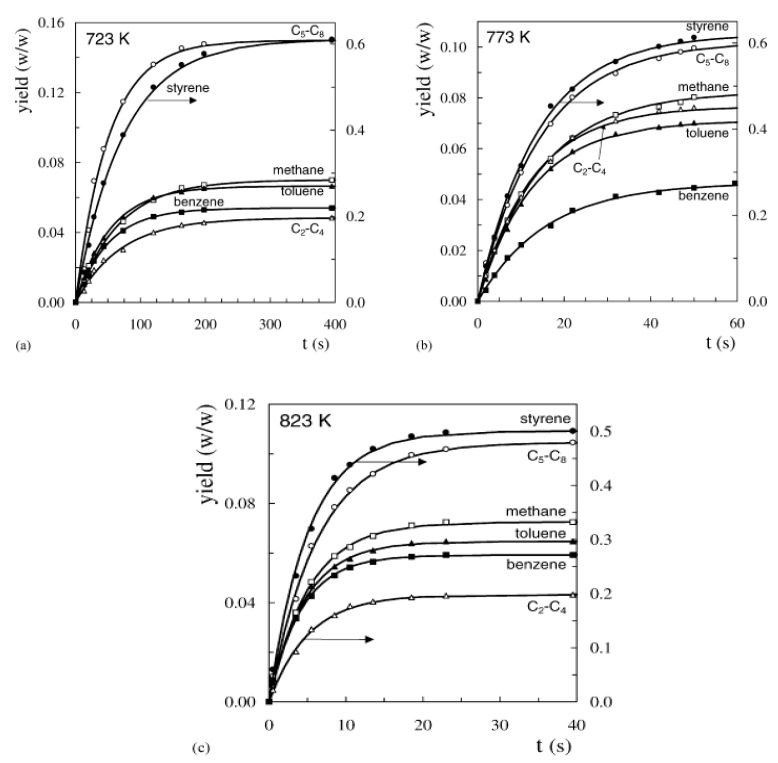
The time-evolution of various yield products of PS pyrolysis carried out in a CSBR at various temperatures: (**a**) 723 K; (**b**) 773 K; and (**c**) 823 K. Reproduce with permission from Aguado et al. [73].

It is noteworthy that the yield of styrene is high with a maximum value of 64.5 wt.% at a temperature of 748 K. Further increasing the temperature to 823 K reduced the styrene yield 50.1 wt.% because of the surge in the production of light olefins by cracking of the aromatic ring. These light olefins are mainly responsible for making up the C_2_–C_4_ lump. Figure 12 displays the trend of time-evolution of individual yield products at various temperatures.

#### 3.2.4. Free-Fall Reactor under Vacuum

**Figure 13:**
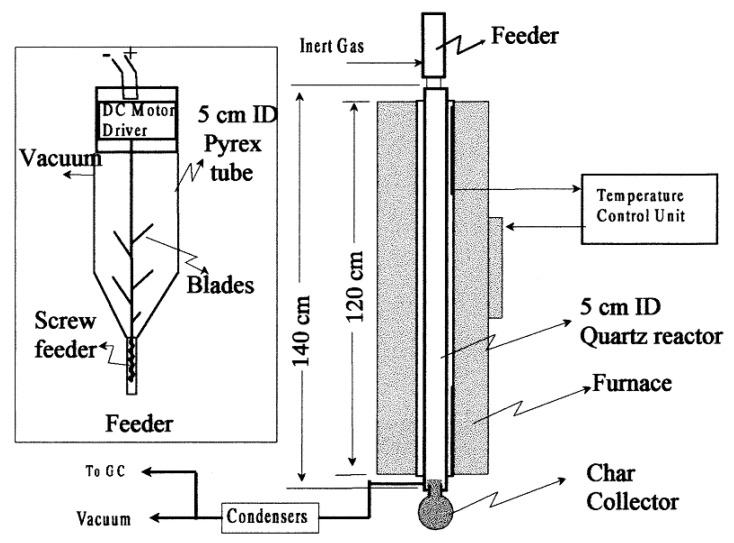
Diagram for free-fall reactor under vacuum. Reproduced with permission from [45].

Karaduman et al. [45] carried out the pyrolysis of polystyrene waste in a free-fall reactor under vacuum conditions. The apparatus for the free-fall reactor is shown in Figure 13. It consists of a quartz reactor jacketed in a hot furnace. A variable speed feeder was installed above the reactor, and a char-collector and two water-cooled condensers were installed below it in series, as displayed in Figure 13. Both the condenser balloon was kept in a salt ice bath. The diameter and height of the reactor were 50 mm and 1400 mm, respectively and the heating zone was 1200 mm in length. To ensure proper feeding of PS particles along the centerline of the reactor and avoid their sticking to the walls, a conical funnel was installed between the feeder and the reactor. The vacuum was needed to extract the waste discharge from the heated zone, thus avoiding unnecessary reactions. The reactor temperature was monitored at two locations 400 and 800 mm from the top by two Ni−CrNi thermocouples. The waste PS foams were cleaned, heated in an oven at 125 °C to make it dense, grounded, and further separated into four size ranges, namely 300–212, 212–50, 150–75 µm, and less than 75 µm diameter employing ISO 3310-1 standard sieves. The feeder was loaded with 75±10 g waste particles of a selected size range and mounted over the pre-heated reactor. The voltage of the DC motor was adjusted to drive the feeder at a feed rate of approximately 2 g/min. Oxygen was expelled from the system by nitrogen. A reduced pressure of 10 kPa was maintained with the help of a vacuum pump. The feeder was started to operate, thereby initiating the reaction that caused the buildup of pressure. After the feedstock was consumed, the vacuum pump was switched off and the system was left to cool down. The final byproducts of the pyrolysis process accumulated in the char collector (solid residue) and the condenser balloons (liquid residue).

A series of experiments were carried out varying the temperature in the range of 700–875 °C to determine the effect of temperature on styrene, benzene, toluene, and naphthalene distribution in the liquid residue and C_1_–C_4_ content in the gaseous residue. The maximum liquid yield was produced at a temperature of about 750 °C and the yield of styrene at about 825 °C. Generally, operating the reactor at higher temperatures reduced the production of solid byproducts while increasing the gaseous residue. Moreover, it was also established that when the reactor was charged with finer feedstock, the total conversion efficiency increased with a higher yield of gaseous products. The pyrolysis in this reactor can be made more efficient if the feedstock was allowed to stay for more time during degradation and removing the primary products from the reactor rapidly, thereby avoiding their further degradation.

**Figure 14:**
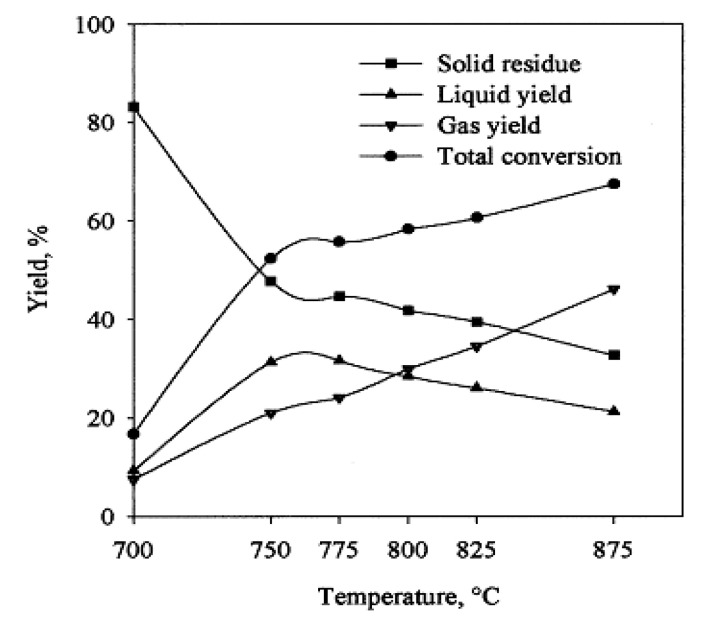
Plot showing the effect of temperature on the product phase yields of PS pyrolysis in a free-fall reactor under vacuum. Reproduced with permission from [45].

Figure 14 displays the effect of temperature on the yields of PS pyrolysis. The yield of gaseous products increased with rising temperatures during the whole temperature range considered. Moreover, with the temperature increase up to 750 °C, the solid yield reduced sharply whereas both the liquid yield and total conversion increased. Beyond 750 °C, the solid yield further decreased albeit at a slower rate. Similarly, the liquid yield also kept reducing, however slowly, probably due to the degradation of expected product styrene.

#### 3.2.5. Microwave-Assisted Pyrolysis

The recent developments in microwave technology have opened a new pathway for the recovery of polystyrene waste through the pyrolysis process. In microwave-assisted pyrolysis, microwave energy absorbing material such as particulate carbon is blended with polystyrene waste. The microwave absorbent material soaks microwave energy to produce sufficient thermal energy that can lead to attaining high temperatures needed to carry out pyrolysis [74]. In contrast to traditional pyrolysis techniques, microwave technology offers various merits like speedy heating, higher production rate, and low production cost. Moreover, unlike conventional pyrolysis techniques, microwave energy is directly supplied to the feedstock material via electromagnetic interaction with the molecules, thus there is no need to squander energy to heat the ambient atmosphere [75]. However, the use of microwave technology has limitations on the industrial scale due to the unavailability of adequate data to quantify the dielectric characteristics of the treated waste stream.

The dielectric property of material governs the efficiency of microwave heating. For example, plastics possess small dielectric constant, and mixing with microwave absorber such as carbon for carrying out pyrolysis may increase the heat absorption rate [74]. Khaghanikavkani [76] proposed that various parameters affect the heating efficiency of a microwave, namely, flow rate of nitrogen, absorber type, and microwave rotation design. The readers are further referred to the following detailed reviews by Fernandez et al. [75], Lam et al. [74], and Undri et al. [77] to understand the issues involved in pyrolysis process based on microwave heating.

### 3.3. Catalysts

#### 3.3.1. Catalyst Importance in Pyrolysis

Catalysts are defined as chemical agents that increase the rate of a chemical reaction while remaining unaltered by the end of the reaction. Catalysts typically reduce the activation energy or alter the mechanism of reaction to increase the reaction rate. In catalytic pyrolysis, the catalyst decreases the operating temperature needed for the pyrolysis and hence reduces the heat energy requirement which favors the industrial application of pyrolysis. Catalysts are extensively employed for pyrolysis in industries and researches to obtain an optimum distribution of end-products and enhance product selectivity. Hence, catalytic pyrolysis is of great importance to get final products of commercial significance like diesel, gasoline, and C_2_–C_4_ olefins that have tremendous demand in the petrochemical industry [48].

#### 3.3.2. Types of Catalysts

Catalysts can be broadly classified into two categories, homogeneous and heterogeneous. Homogeneous catalysts are those which have the same phase as that of reactants, while heterogeneous catalysts are those which are in a different phase than that of the substrate, normally solid and gas, respectively. Typically, heterogeneous catalysts are mostly employed in pyrolysis as the liquid product mixture can be separated from the catalyst in solid-phase without any difficulty. Heterogeneous catalysts are also preferred from the economic viewpoint since catalysts are costly and hence their reuse is highly desirable. A heterogeneous catalyst is categorized as basic oxides, conventional acid solid, nanocrystalline zeolites, mesostructured catalyst, and metal supported on carbon [78].

##### Bentonite Catalyst

Dewangga et al. [79] employed bentonite clay as a catalyst to investigate its effect on catalytic pyrolysis of polystyrene waste. They mixed 100 g of polystyrene waste with bentonite powder having 0%, 5%, 10%, 15%, 20%, and 25% of polystyrene weight. The pyrolysis was carried out in a batch reactor with a maximum temperature of 400 °C and was held at this temperature for 60 min. The liquid product was analyzed employing Gas chromatography-mass spectrometry (GC–MS) to ascertain the percentage of components in the product, as shown in Table 6. The production of liquid products in the absence of catalyst was 37.89%. With the increase of catalyst weight, the liquid product yield increased, reaching a maximum of 74.55% at 25% catalyst by weight. The liquid analysis showed that styrene compounds (C_8_H_8_) toluene and benzene were the chief components in the liquid product.

##### Zinc Bulk Catalysts

**Figure 15:**
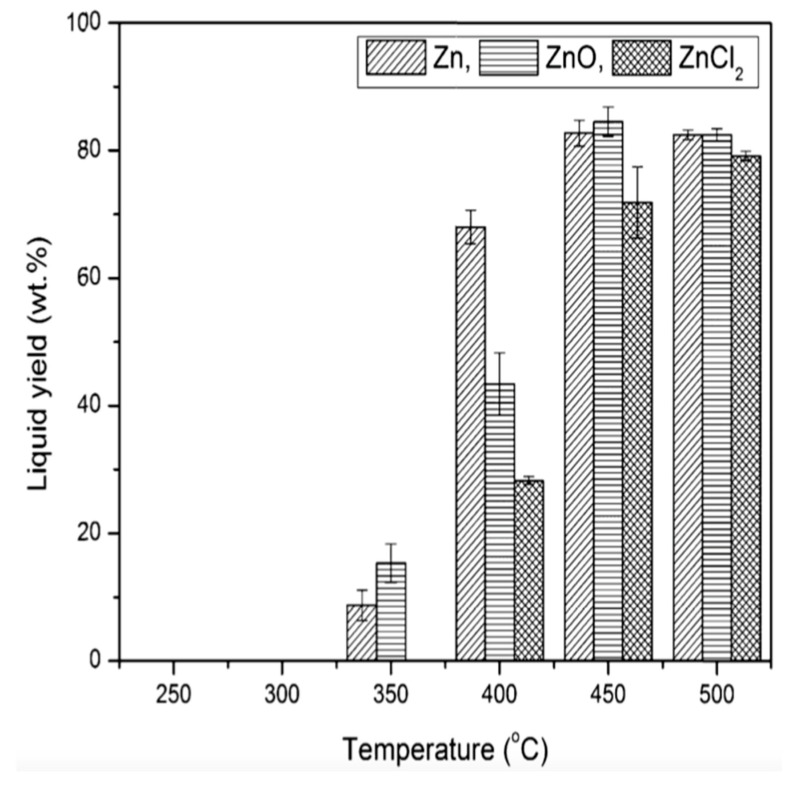
Effect of temperature on the liquid product yield in pyrolysis of EPSW employing $\mathrm{Zn},{\;\mathrm{ZnO}\;\mathrm{and}\;\mathrm{ZnCl}}_{2}$ catalyst with 60 min heating time and 1:0.2 feed to catalyst ratio [53].

Adnan et al. [53] employed zinc bulk catalysts ($\mathrm{Zn},{\;\mathrm{ZnO}\;\mathrm{and}\;\mathrm{ZnCl}}_{2}$) for carrying out the catalytic pyrolysis of expanded polystyrene waste (EPSW). The catalysts were found to be highly active and provided better selectivity of end products. The catalysts were used in the form of granules with Zn granules (99.9%), ZnO (99.0–100.5%), and ${\mathrm{ZnCl}}_{2}$ (99%). The pyrolysis was carried out in an efficient Pyrex batch reactor. Out of the various zinc bulk catalysts, the Zn metal catalyst performed best for the economical pyrolysis of EPSW. The pyrolysis produced 96.73 ± 0.12 wt.% liquid products with 2.47 wt.% toluene, 1.16 wt.% ethylbenzene, 47.96 wt.% styrene monomer, and 1.90 wt.% α-Methylstyrene. The holding time was 120 min at temperature 450 °C and 1:0.2 feed to catalyst ratio. The liquid components were separated by fractional distillation process following PN-81/C-04012 method based on the boiling point of the products. To study the effect of pyrolysis temperature on the yield of liquid products, the temperature was varied from 250–500 °C for all the three catalysts with 60 min heating time and 1:0.2 feed to catalyst ratio. The variation of liquid yield with varying temperatures for all three catalysts is shown in Figure 15.

##### FCC Catalyst

Fluid catalytic cracking (FCC) catalyst has four main components: crystalline zeolite, matrix, filler, and binder. Zeolite is the active element and can contribute around 15 to 50 weight percent of the catalyst. The zeolite Faujasite is used in FCC units. The zeolite is a strong acid and does not dissolve in the reaction medium. The alumina matrix element of the FCC catalyst is also responsible for catalytic activity sites. The filler and binder elements furnish physical strength and integrity to the FCC catalyst. Typically, the binder and filler are silica sol and clay (kaolin), respectively. The FCC catalysts are in the form of a fine powder having a bulk density of 0.80 to 0.96 g/cm3 and particle size distribution ranging from 10 to 150 µm [80]. They are generally employed in the petroleum refineries to convert high molecular weight hydrocarbon fractions of crude petroleum oil into more useful gasoline and liquid petroleum gas (LPG) fractions [81]. The FCC catalyst that had already been used is generally known as ‘spent FCC catalyst’ and obtained from the FCC process in petroleum refineries. It comes in the commercial market with various degrees of contamination and can be utilized again in the pyrolysis process.

Kyong et al. [62] studied the pyrolysis of High-Density Polyethylene (HDPE), Low-density polyethylene (LDPE), Polypropylene (PP) and Polystyrene (PS) in the presence of spent FCC catalyst in a stirred semi-batch reactor at 400 °C. They mixed 20 g of catalyst in 200 g of feedstock and provided heat at a rate of 7 °C/min. All types of plastics yielded liquid oil exceeding 80 wt.% with PS producing the highest amount of around 90 wt.%. The number of liquid product yields based on the type of plastics was obtained in the order PS > PP > PE (HDPE, LDPE), while the gaseous product yield followed the order PE > PP > PS. In conclusion, the spent FCC catalyst exhibited high catalytic efficiency with the liquid products yields above 80 wt.% for the three types of plastics. Moreover, the FCC catalyst is economical since it is a ‘recycled’ catalyst.

##### Acidic and Basic Catalysts

Anwar et al. [82] depolymerized PS waste into styrene utilizing several acidic and basic catalysts. The pyrolysis was carried out under atmospheric pressure at a temperature range of 300–350 °C. They found calcium oxide to be the most economical and efficient catalyst among the metal oxides to depolymerize waste PS into styrene with 77% distillate recovery. Metal carbonates produced pure styrene, although the yield was less. Ukie et al. [19] reported that acidic catalysts exhibited poor efficiency in depolymerization as compared to solid base catalysts, due to the generation of carbocations that further leads to the synthesis of various side products like toluene, benzene, ethylbenzene, indane derivatives, and coke. These side products are formed spontaneously due to very strong interaction between styrene or its precursor with acidic catalysts.

Anwar et al. [82] also carried out the catalytic pyrolysis of PS using various metal oxides in paraffin oil. However, they did not notice any significant difference in the yield of styrene. They also incorporated some transition metal oxides as catalysts as summarized in Table 7, but the product yields were poor as compared to alkaline earth metal oxides.

In the category of solid acidic and basic catalysts, such as magnesium oxide (MgO), calcium oxide (CaO), potassium oxide (KO), zinc oxide (ZnO), copper oxide (CuO), barium oxide (BaO), iron oxide (FeO), chromium oxide (CrO), cobalt oxide ($Co_{3}O_{4}$), titanium dioxide ($TiO_{2}$), silica-alumina ($SiO_{2}/Al_{2}O_{3}$), zeolite (HZSM5), and active carbon (AC), the catalyst BaO was reported to be the most efficient to degrade PS into monomer and dimer at [19,30]. Shah et al. carried out the pyrolysis of PS in a batch reactor employing Mg, MgO and ${\mathrm{MgCO}}_{3}$ as catalysts [83], and also compared their results with previous studies of Ukei et al. and Audisio et al. [19,84], as shown in Table 8. Their results also corroborated the previous finding about the high effectiveness of BaO.

Furthermore, in a more recent study by Tiwary et al. showed that alkaline oxide is more reactive towards PS in comparison to transition metal oxides, except MgO which exhibited higher reactivity because of the formation of stable manganese (II) oxide during the pyrolysis [85].

### 3.4. Effect of Solvent

Pyrolysis process can also be carried out at comparatively low temperature range of 300–450 °C by elevating the ambient pressure of the reactor vessel. However, this process requires the addition of solvents. Murakata et al. demonstrated for the first time, the advantages of pyrolysis of PS in solution as compared to the conventional one [86]. They employed 2-Naphthol, phenol, l-methylnaphthalene, decalin, diphenylamine, tetralin, and 9,10-dihydroanthracene as solvents, all of them were reagent grade. These solvents were chosen due to their superior thermal characteristics, such as better thermal stability at reaction temperatures and comparatively low vapor pressure. They discussed the degradation mechanism and reported the conversion efficiency of the pyrolysis in various solvents, as detailed in Table 9.

However, recently researchers also employed water as a solvent and when it is utilized then the pyrolysis is called hydrothermal pyrolysis [87,88]. Nevertheless, due to its high critical points and corrosive nature it produces less oil, and the final product achieved turns out to have high oxygenated compounds and high viscosity [89,90]. It is communicated in the previous literature that a good solvent should not be corrosive, can be conveniently separated from final product, has low critical points and augment the produced oil quality and quantity [91].

Shin et al. carried out pyrolysis of PS employing water, n-hexane and methanol under supercritical settings and found that the process utilizing water as a solvent showed the greatest activation energy of 157 kJ/mol, while the process employing n-hexane and methanol reported activation energy of 132 kJ/mole and 117.2 kJ/mole, respectively [92]. This suggests that organic solvents have higher efficiency than water.

Some scientific studies of pyrolysis of PS employing various solvents explored the influence of reaction time on the yield of oil [92,93]. The studies showed that prolonging the time of reaction increased production of oil. Nevertheless, further extending the time of reaction did not result in any significant improvement in the yield of oil. Furthermore, Hwang et al. utilized n- hexane as a solvent and showed that, within the temperature range of 370–390 °C, the oil yield remained unaltered even after 0.5 h, suggesting the attainment of reaction equilibrium [13].

Likewise, some researchers employed alcoholic solvents for pyrolysis of PS waste. They established that alcoholic solvents act as better hydrogen donors resulting in moderate process conditions for converting carbonaceous raw matter into liquid fuel [94,95]. Moreover, alcoholic solvents are characterized by lower critical points which make them more efficient than subcritical water [96]. Past research also suggested that the employment of alcoholic solvents enhanced the dissolution and stabilization of the intermediate reaction products, eventuating higher conversion rates with higher oil production [97,98].

**Figure 16:**
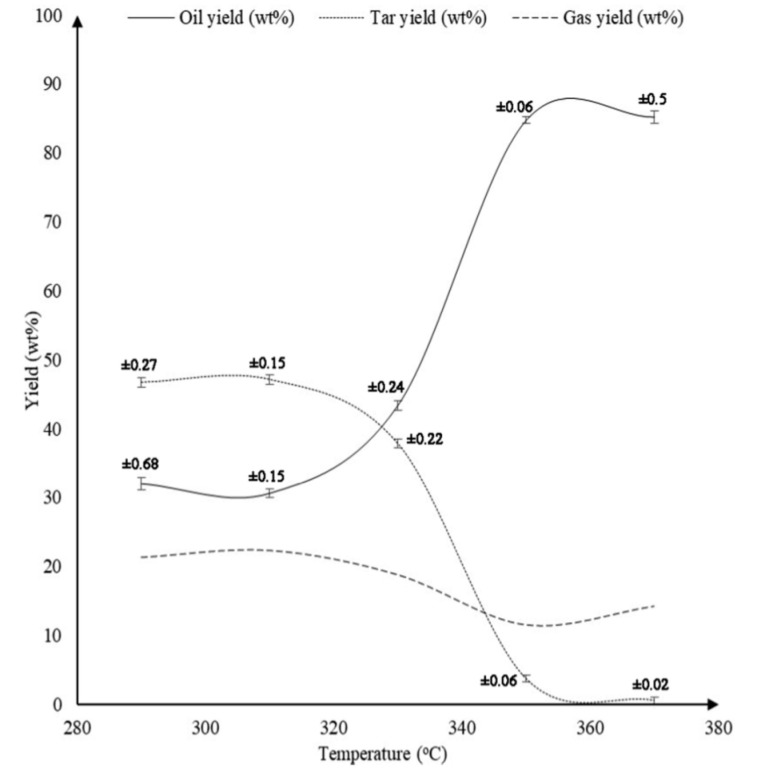
Effect of temperature on the product yields [41].

**Figure 17:**
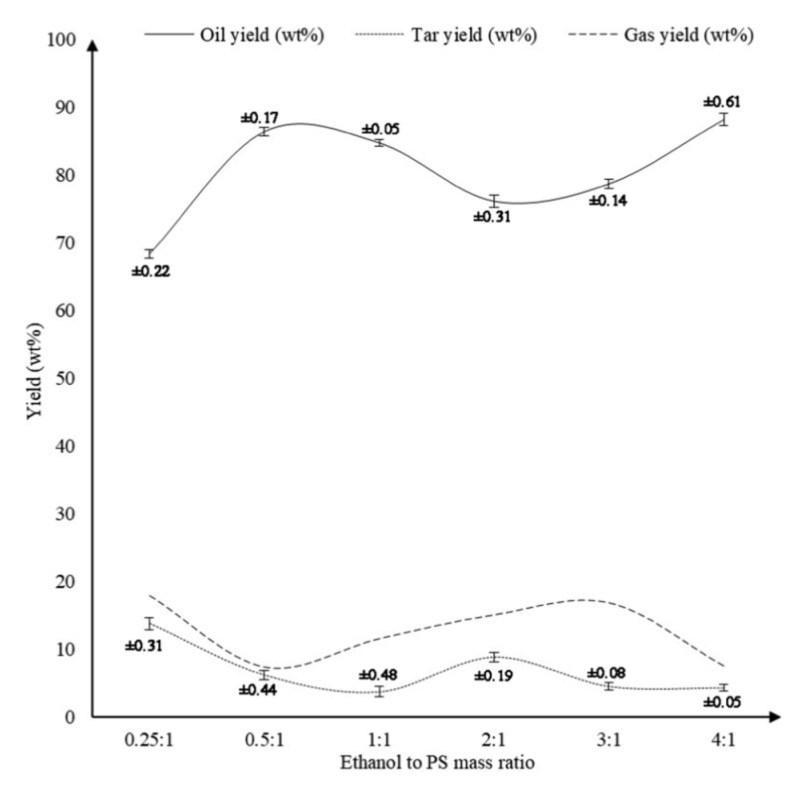
Effect of ethanol to PS mass ratio on the product yields [41].

**Figure 18:**
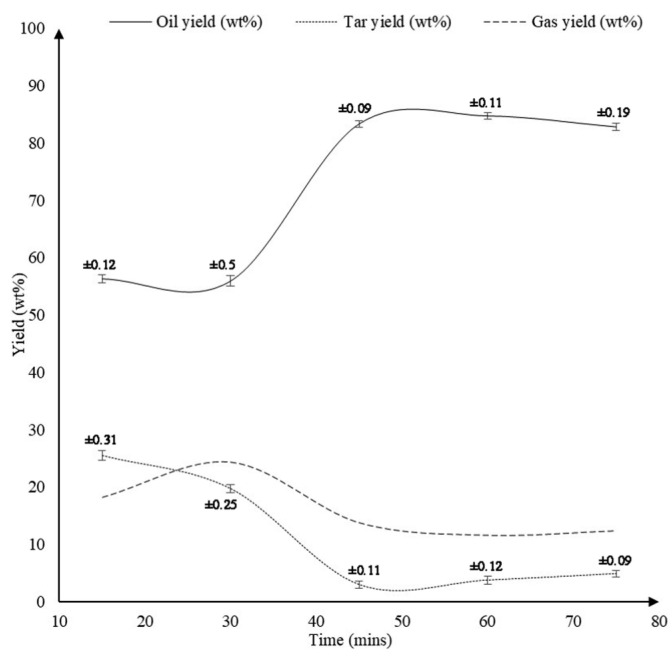
Effect of reaction time on the product yields [41].

A more recent study by Ahmad et al. [41] employed ethanol as a solvent. They studied the effect of temperature, ethanol to PS mass ratio and time of reaction on the quality and quantity of final products. The pyrolysis was carried out at temperatures of 290, 310, 330, 350, and 370 °C while maintaining ethanol to PS mass ratio and reaction time constant at 1:1 and 60 min respectively. Figure 16 shows that as the temperature was elevated from 290 °C to 370 °C, the oil production was enhanced from 32.0% to 85.2%. A remarkable increase of oil yield was observed as the temperature was increased from 330 °C to 350 °C, wherein oil produced amplified from 43.4% to 84.7%. Further increasing the temperature beyond 350 °C did not show any significant improvement in the production of oil. Ahmad et al. also studied the effect of ethanol to PS mass ratio by varying it as 0.25:1, 0.5:1, 1:1, 2:1, 3:1, and 4:1 while maintaining the temperature and time of reaction to be constant at 350 °C and 60 min respectively. Figure 17 shows that increasing the ethanol to PS mass ratio from 0.25:1 to 0.5:1 significantly increased the yield of oil from 68.4% to 86.4%. This result is even expected since the presence of ethanol in greater quantity assists the liquefaction process. Nevertheless, further increasing ethanol to PS mass ratio from 0.5:1 to 3:1 curtailed the production of oil to 78.7%, while the gas production increased from 7.4% to 16.8%. The effect of varying the time of reaction as 15, 30, 45, 60, and 75 min while maintaining the temperature and ethanol to PS mass ratio constant at 350 °C and 1:1 respectively is displayed in Figure 18. It can be observed that below 30 min duration of reaction the oil produced is 56% which is a poor outcome. This is due to the fact that the system did not get enough time to complete the reaction. Nevertheless, when the reaction duration was increased from 30 min to 45 min, oil production amplified significantly from 55.9% to 83.3%. The highest oil yield of 84.7% was attained when the duration of reaction was set to 60 min. Further increasing the reaction time did not show any significant improvement in the yield of oil.

## 4. Co-Pyrolysis of Polystyrene

To improve the yield of liquid products and achieve a better product selectivity with superior quality, co-pyrolysis of polymers with coal has been proposed by Straka et al. [99]. Based on this finding, Hussain et al. demonstrated the co-pyrolysis of PS with coal leveraging the advantage of high temperature achieved by interaction of microwave with copper [100]. In general, microwave-assisted pyrolysis is carried out by either employing microwave-absorbing materials [101] or microwave-induced plasmas [102]. Hussain et al. conducted the co-pyrolysis of PS and coal in a microwave placed in a clay reactor equipped with copper coil. The copper coil produced a very high amount of heat, raising the temperature approximately in the melting range of copper, within a very short duration. Table 10. details the comparison of pyrolysis of pure coal and co-pyrolysis of coal with PS. It can be observed that the co-pyrolysis of coal with PS enhances the liquid fuel yield, because of the interaction of active species with the coal.

It has been observed by many researchers that co-pyrolysis of biomass with polymers minimized coke formation due to the development of synergic effects [103,104]. When only biomass is pyrolyzed, the obtained liquid products are usually oxygenated and unstable. These biofuels cannot be directly utilized because of their poor calorific value, high viscosity, reactivity and acidity. Thus, they have to be upgraded before use. However, if biomass is co-pyrolyzed with polymers, synergy evolves due to the rich hydrogen contents of the polymers during degradation process [105,106,107]. The co-pyrolysis of biomass and PS has also been demonstrated by many researchers [107,108,109]. Abnisa et al. showed the results of co-pyrolysis of palm shell with PS and compared the output with the pyrolysis of palm shell alone, as detailed in Table 11. They reported that the oil yield from palm shell pyrolysis was about 46.13 wt.%, which increased to 61.63% when palm shell was co-pyrolyzed with PS (50:50 wt.% ratio).

## 5. Patents Invented in the Field of Polystyrene Pyrolysis

In this section, we will review the various patents invented by scientists in the field of PS pyrolysis. A patent invented by White et al., disclosed a method wherein PS wastes in solid and/or liquid form are blended with a binder, and then converted into the pellet form [110]. These pellets were fed into a furnace comprising of a pyrolysis zone, wherein they get partly gasified. The solid residues were transferred to the oxidation zone of the furnace prior to entering into sintering zone, whereas liquid products were allowed to condense. Later on, a technique for decomposing PS directly into styrene monomer was patented by Northemann [111]. The PS waste in liquid and/or solid state was pyrolyzed in a fluidized bed reactor within a temperature range of 400–700 °C. To amplify the heat transfer rate a magnesium or aluminum silicate medium was used. The PS was depolymerized in the reactor at a mean residence time of less than a minute. The styrene was then recovered from the gaseous cracking products. Likewise, Bouziane disclosed a pyrolysis batch process for recycling PS to produce useful light oil and fuel gases [112]. The charge is fed into a rotatable reactor which is evacuated, rotated and heated till the initiation of exothermic reaction. The reactor pressure is then increased to the atmospheric pressure and above, as per the need. The continuous rotation and heating produced condensable hydrocarbon vapors and gaseous hydrocarbons. The vapors are allowed to condense into oil while the gaseous hydrocarbons were used as a fuel for heating the reactor. Matsubayashi, disclosed the recovery of styrene from PS in a solvent, wherein a solution containing polystyrene is supplied to a pyrolysis device in which the solution is heated to a temperature at which the polystyrene is cracked and thus styrene is obtained [113].

Yang invented a patent on catalytic cracking, wherein he mixed the waste PS with a catalyst such as ZSM-5 and fed the charge into a reactor for catalytic cracking reaction [114]. The temperature was maintained in the range of 280–480 °C and the solid impurities were removed from the generated vapor. The vapor was condensed in a condenser, and the condensate was distilled and separated to obtain gasoline and diesel oil. Carner, however, employed other catalysts comprising of iridium, manganese, gold, silver or other metals and their oxides [115]. The PS waste was blended with the catalyst and a reaction fluid, such as oil. The feedstock is then converted into slurry and subsequently heated in a evacuated environment such that the individual macromolecules of PS decompose into their monomers. Srinakruang, through his patent demonstrated the use of dolomite catalyst, within a temperature range of 330–400 °C [116]. The resulting liquid is subjected to a catalytic cracking reaction in a semi-batch reactor, producing superior quality fuel, predominantly light and heavy naphtha.

DeWhitt, invented a patent in which he treated the PS waste in a pyrolysis chamber within a temperature range of 270–375 °C [117]. The chamber is gradually heated in steps, and the vapor of pyrolyzed inorganic species and gaseous organic species was removed by a vacuum. The vapor was then allowed to get in contact with a pH buffered aqueous media which resulted in the condensation of gaseous organic species. The inorganic components, namely chlorine and bromine were separated from the oil products. McNamara et al. pyrolysed PS in an oxygen-free atmosphere to produce pyrolysis gases [118]. These gases were allowed to get in contact with plates of a contactor vessel, such that long-chain gas components condense and re-pyrolyzed to achieve thermal degradation. On the other hand, short-chain gas components leave out the contactor vessel generating one or more on-specification fuel products. A patent invented by Bordynuik, disclosed a novel reactor for carrying out pyrolysis of PS within a temperature range of 340–445 °C, by cracking the hydrocarbons present in it [119]. The PS is converted into vapours comprising hydrocarbon compounds. The hydrocarbon substituents were separated based on their boiling points via separating vessels, and condensed into different petroleum products (diesel, gasoline, furnace fuel, kerosene, propane, butane, ethane, and methane, etc.). The fuels produced during the process are recyclable for upstream purpose.

## 6. Recommendations

Pyrolysis of PS turns out to be a robust and efficient technique to produce a variety of beneficial hydrocarbons that can be utilized as chemical feedstocks or an alternative to non-renewable fossil fuels. Pyrolysis is eco-friendly as compared to other treatment methods, since it is carried out in an oxygen-free atmosphere, thus eliminating the production of dioxins, and minimizing the carbon monoxide and dioxide emissions. It is also a pliable technique which can easily incorporate the variation in temperature, pressure, and residence time to obtain desired end-products. The pressure governing pyrolysis can be a playing factor at lower temperatures. Nevertheless, the influence of pressure on pyrolysis is not well studied as compared to other parameters, like temperature. This parameter calls for attention from the researchers to be investigated, so that a viable solution of the limitation in achieving high operating temperatures during pyrolysis can be realized.

Several types of reactors have been considered by researchers for carrying pyrolysis. Although, batch reactors offer a high conversion efficiency, they have been found to be inconsistent and uneconomical due to the requirement of recurrent feedstock charging ultimately shooting up the labour cost in the process. Therefore, a continuous pyrolysis reactor is recommended for large scale operations. The conical spouted bed reactor (CSPR) is a good option when large particles with density disparity have to be handled, whereas microwave-assisted reactor is a good alternative for reducing heating time and operational cost. Moreover, fixed-bed reactor will be perfect to be used as a secondary pyrolysis unit to treat the by-products of the first reactor. In this way, one can tailor the product distribution as per the needs very easily.

It is always recommended to carry out thermal pyrolysis near oil refineries, since its end-products need further upgradation. Catalytic pyrolysis presents several advantages over the thermal pyrolysis, because it decreases the residence time of the process and fine-tune the product selectivity. Moreover, employing catalysts like zeolites produce high quality end-products from the class of automobile engine fuels, thus eliminating any further need of upgradation in the downstream of pyrolysis process. Nevertheless, catalysts are costly, and any risk of catalytic poisoning and its deactivation from the feedstock impurities have to be considered well in advance.

The pyrolysis process has the proven ability to convert PS waste into liquid fuels. However, the economic assessment of this process at a pilot scale is recommended to facilitate fuel production at an industrial scale. The agenda of developing this technology should also be included in the national policies of developing nations which currently do not own the required infrastructure to collect, separate and recycle PS waste. An effective collection strategy is vital to supply a continuous stream of PS waste to the reactor; thus governments should focus on promoting efficient collection schemes in order to advance this technology at a larger scale.

## 7. Conclusions

Nowadays, the most customary exercise to dispose polystyrene waste is landfill which is infelicitous because of its very slow decomposition rate posing severe environmental risks. In such a scenario, pyrolysis of PS appears to be the best alternative where PS gets converted into useful products. Moreover, the pyrolysis process is also favorable as it can diminish our reliance on fossil fuels which are non-renewable energy. This review article has presented a comprehensive survey of the various process parameters involved and their effect on the pyrolysis of PS. The paper discusses the experimental results of pyrolysis process from various research groups that employed different temperatures, reactors and catalysts to optimize the liquid oil yield. The pyrolysis can be performed in both thermal and catalytic environment, however the catalytic process requires lower operating temperatures with a higher production of liquid oil. The experimental studies discussed suggest that the operating temperature of 425 °C is optimum for PS pyrolysis. At this temperature, maximum quantity of liquid oil is yielded. However, the optimal temperature basically depends on the type of by-products we wish to obtain. If liquid oil is preferred, then a relatively lower temperature range of 300–500 °C is recommended while if we want gaseous or char as by-products then a temperature beyond 500 °C is preferred.

The choice of catalysts plays an important role on the yield of pyrolysis by-products. Bentonite catalysts produced mainly styrene compounds toluene and benzene among the liquid product. Zinc bulk catalysts were highly active and offered better selectivity of end products. The spent FCC catalyst displayed high catalytic efficiency with the liquid products yields above 80 wt.% for the three types of plastics. Furthermore, the FCC catalyst is cheaper as it is a ‘recycled’ catalyst. Out of metal oxides, calcium oxide turned out to be economical and effective catalyst to depolymerize waste PS into styrene with 77% distillate recovery. Acidic catalysts displayed penurious efficiency in depolymerization in comparison to solid base catalysts as they generate side products.

The utilization of solvent for performing pyrolysis process showed that the highest yield of oil occurs at 350 °C, Ethanol/PS ratio of 0.5:1, and reaction time of 60 min. The GC–MS analysis revealed the presence of alkenes, aromatics, and alkyl compounds in the oil signifying that it can be a promising source of fuel. In conclusion, future research directions regarding pyrolysis of PS should include the development of a better understanding of the interplay between the temperature, catalysts, reactors, and solvents, and establishing a better cognizance of the influence of these parameters to realize optimum reactor designs.

## Figures and Tables

**Table 1:** Advantage of various methods employed for PS pyrolysis.

S. No.	Methods for Polystyrene Pyrolysis	Advantages	Ref.
1.	Classical Heating Method		
	i. Batch Reactor	high conversion efficiency	[42]
	ii. Fixed Bed Reactor	simplicity in design	[43]
	iii. Fluidized Bed Reactor	(a) yield of liquid products is more than 90wt.%. (b) formation of gas and coke is relatively insignificant.	[12]
	iv. Conical Spouted Bed Reactor	(a) allows better blending of charge (b) can treat large particles with density disparity	[44]
	v. Free-fall Reactor Under Vacuum	produces important liquid chemicals such as benzene, toluene, and naphthalene besides styrene monomer and valuable gaseous output.	[45]
2.	Microwave-Assisted Pyrolysis	(a) extremely rapid heating (b) higher production rate (c) low production cost (d) energy saving	[46]
3.	Catalytic Pyrolysis	(a) decreases the operating temperature needed for the pyrolysis (b) reduces the heat energy requirement (c) favors the industrial application of pyrolysis (d) produce final products of commercial significance	[47,48]
4.	Solvent-Assisted Pyrolysis	(a) high heat and mass transfer rates (b) reduces operating temperature (c) higher liquid yields	[49,50]

**Table 2:** Yields of oil and gaseous products in PS isothermal pyrolysis at different temperatures [29].

Reaction Temperature (°C)	Yield (wt.%)	
	Oil	Gas
370	96.40	3.60
380	96.10	3.90
390	95.90	4.10
400	94.80	5.20

**Table 3:** Summary of PS pyrolysis in batch reactors.

Reactor	Process Parameters				Yield (wt.%)			Additional Information	Ref.
	Temp. (°C)	Pressure	Heating Rate (°C/min)	Duration (min)	Oil	Gas	Solid		
Batch	581	-	-	-	89.5	9.9	0.6	64.9 wt.% of liquid comprised of styrene	[26]
Batch	500	-	-	150	96.7	3.27	0	used Zn catalyst, cat./PS = 5 *w*/*w*	[53]
Semi-batch	400	1 atm	7	-	90	6	4	used FCC catalyst, cat./PS = 10 *w*/*w*, stirring rate of 200 rpm	[62]
Pressurized batch	425	0.31–1.6 MPa	10	60	97	2.50	0.5		[25]

**Table 4:** Main products of PS pyrolysis [12].

Number P	Pyrolysis Products	Fractions
1	Benzene	Low boiling fraction (G1)
2	Toluene	
3	Ethylbenzene	
4	Xylene	
5	Styrene (Monomer)	
6	α-Methylstyrene	
7	Others	
8	1,2-Diphenylethane	Medium boiling fraction (G2)
9	1,3-Diphenylpropane	
10	2,4-Diphenyl-1-butene (Dimer)	
11	2,4-Diphenyl-1-pentene	
12	Others	
13	2,4,6-Triphenyl-1-hexene (Trimer)	High boiling fraction (G3)
14	Others	

**Table 5:** Yields (wt.%) of the products obtained by polystyrene pyrolysis in the CSBR at different temperatures [73].

Compound	723 K	748 K	773 K	823 K
Methane	7	8.30	8.30	7.30
C_2_–C_4_	4.80	5.10	7.70	19.80
C_5_–C_8_	15	11.30	10.20	10.40
Benzene	5.40	3.80	4.60	5.90
Toluene	6.70	7	7.10	6.50
Styrene	61.10	64.50	62.10	50.10

**Table 6:** Effect of catalyst ratio on product yield at 400 °C [79].

Catalyst: Polystyrene Ratio (%)	Contents of Products (wt.%)		
	Liquid	Residue	Gas
0	37.89	3.66	58.45
5	47.93	2.94	49.12
10	62.16	2.85	34.99
15	70.42	2.80	26.77
20	74.10	2.57	23.34
25	74.55	2.24	23.21

**Table 7:** Catalytic depolymerization of polystyrene [82].

Catalyst/Solvent	Catalyst: Polystyrene	Distillation Range (°C)	Distillate Yield (%)	Refractive Index	Purity of Styrene (%)
MgO	1:10	142–144	40	1.5465	91.5
CaO	1:8	142–144	77	1.5465	91.5
${\mathrm{MnO}}_{2}$	1:10	142–144	60	1.5466	91.7
ZnO	1:10	142–144	34	1.5465	91.5
${\mathrm{Al}}_{2}O_{3}$	1:10	142–144	56	1.5464	91.4
${\mathrm{SiO}}_{2}$	1:10	142–144	56	1.5464	91.4
${\mathrm{ZnCl}}_{2}$	1:8	140–142	42	1.5465	91.0
${\mathrm{AlCl}}_{3}$	1:10	140–142	23	1.5464	91.0
PTSA	1:100	120	31	1.5420	-
Acetic acid	1:25	138–140	30	1.5425	-
Formic acid	1:25	138–140	33	1.5408	-
China clay	1:10	134–138	35	1.5406	-
Bentonite	1:10	120–130	45	1.5400	-
${\mathrm{Na}}_{2}{\mathrm{CO}}_{3}$	1:20	144	52	1.5466	92.0
${\mathrm{NiCO}}_{3}$	1:20	144	56	1.5466	92.0
${\mathrm{CaCO}}_{3}$	1:20	144	56	1.5466	92.0
$K_{2}{\mathrm{CO}}_{3}$	1:30	144	56	1.5466	92.0
${\mathrm{BaCO}}_{3}$	1:20	144	49	1.5466	92.0
Mg/paraffin oil	1:10	142–144	41	1.5464	91.5
CaO/paraffin oil	1:10	142–144	74	1.5464	91.5
${\mathrm{MnO}}_{2}$/paraffin oil	1:10	142–144	36	1.5465	91.7
ZnO/paraffin oil	1:10	142–144	48	1.5465	91.7
${\mathrm{Al}}_{2}O_{3}$/paraffin oil	1:10	142–144	49	1.5465	91.7
${\mathrm{SiO}}_{2}$/paraffin oil	1:10	142–144	26	1.5464	91.5

**Table 8:** Comparison of reaction conditions and their end-products [83].

Catalysts	Thermal	Mg	MgO	$MgCO_{3}$	BaO	$SiO_{2}/Al_{2}O_{3}$
*Reaction conditions*						
Temperature (°C)	500	450	400	400	450	400
Time (min)	150	30	120	120	180	120
PS to Cat. ratio	-	1:0.3	1:0.3	1:0.3	-	-
*Contents of products (wt.%)*						
Liquid yields	78.07	82.20	91.60	81.80	93.40	83.5
Gas and cokes yield	20.40	17.20	7.00	13.07	-	11.70
Residue	1.53	0.60	1.40	5.13	3.20	4.80
*Contents of liquid (wt.%)*						
Benzene	0.06	0.06	0.08	-	-	1.90
Toluene	2.64	2.73	4.05	6.28	1.60	4.70
Ethylbenzene	1.09	0.88	2.23	7.59	0.20	22.60
Styrene	50.36	66.56	54.53	55.23	76.40	36.00
Isopropylbenzene	0.06	0.34	0.15	1.28		0.04
α-Methylstyrene	17.02	1.52	3.74	5.72	1.40	7.60
1,3-Diphenyl-propane	2.53	2.42	5.40	6.30	18.30	-
Other	26.24	25.49	29.82	17.60	2.10	27.16

**Table 9:** Conversion efficiency of PS in various solvents at 400 °C [86].

Solvent	Conversion Efficiency (%)
2-Naphthol	100
Phenol	87.8
1-Methylnaphthalene	84.2
Decalin	73.8
Diphenylamine	70.6
Tetralin	50.6
9,10-Dihydroanthracene	43.3

**Table 10:** Comparison of pyrolysis of pure coal and co-pyrolysis of coal with polystyrene [100].

Type of Reaction	Time (min)	% Aqueous Liquid	% Liquid Oil	% Sticky Liquid	% Gases	% Residue
Coal co-pyrolysis with polystyrene	16	10.00 ± 0.04	66.00 ± 0.03	4.00 ± 0.03	6.00 ± 0.03	18.00 ± 0.03
Coal pyrolysis	18	25.0 ± 0.03	44.0 ± 0.02	7.0 ± 0.03	6.0 ± 0.01	18.0 ± 0.03

**Table 11:** Product distribution from the co-pyrolysis of palm shell and polystyrene at various ratios [108].

Polystyrene Feed (%)	Pyrolysis Oil (wt.%)	Char (wt.%)	Non-Condensable Gases (wt.%)
20	47.73	27.81	24.46
30	49.93	23.22	26.85
40	59.13	20.18	20.69

## Data Availability

The data presented in this study are available on request from the corresponding author.
